# Quantifying internal intervertebral disc strains to assess nucleus replacement device designs: a digital volume correlation and ultra-high-resolution MRI study

**DOI:** 10.3389/fbioe.2023.1229388

**Published:** 2023-10-02

**Authors:** Tamanna Rahman, Saman Tavana, Nicoleta Baxan, Kay A. Raftery, George Morgan, Thomas P. Schaer, Nigel Smith, Axel Moore, Jonathan Bull, Molly M. Stevens, Nicolas Newell

**Affiliations:** ^1^ Department of Bioengineering, Imperial College London, London, United Kingdom; ^2^ Department of Mechanical Engineering, Biomechanics Group, Imperial College London, London, United Kingdom; ^3^ Biological Imaging Centre, Central Biomedical Services, Imperial College London, London, United Kingdom; ^4^ Faculty of Medicine, National Heart and Lung Institute, Imperial College London, London, United Kingdom; ^5^ Department of Clinical Studies, New Bolton Center, School of Veterinary Medicine, University of Pennsylvania, Kennett Square, PA, United States; ^6^ Division of Surgery and Interventional Science, University College London, Stanmore, United Kingdom; ^7^ Department of Materials and Institute of Biomedical Engineering, Imperial College London, London, United Kingdom; ^8^ Neurosurgery, BARTS Health NHS Trust, London, United Kingdom

**Keywords:** nucleus replacement surgery, IVD strains, digital volume correlation, nuclectomy, nucleus replacement device

## Abstract

**Introduction:** Nucleus replacement has been proposed as a treatment to restore biomechanics and relieve pain in degenerate intervertebral discs (IVDs). Multiple nucleus replacement devices (NRDs) have been developed, however, none are currently used routinely in clinic. A better understanding of the interactions between NRDs and surrounding tissues may provide insight into the causes of implant failure and provide target properties for future NRD designs. The aim of this study was to non-invasively quantify 3D strains within the IVD through three stages of nucleus replacement surgery: intact, post-nuclectomy, and post-treatment.

**Methods:** Digital volume correlation (DVC) combined with 9.4T MRI was used to measure strains in seven human cadaveric specimens (42 ± 18 years) when axially compressed to 1 kN. Nucleus material was removed from each specimen creating a cavity that was filled with a hydrogel-based NRD.

**Results:** Nucleus removal led to loss of disc height (12.6 ± 4.4%, *p* = 0.004) which was restored post-treatment (within 5.3 ± 3.1% of the intact state, *p* > 0.05). Nuclectomy led to increased circumferential strains in the lateral annulus region compared to the intact state (−4.0 ± 3.4% vs. 1.7 ± 6.0%, *p* = 0.013), and increased maximum shear strains in the posterior annulus region (14.6 ± 1.7% vs. 19.4 ± 2.6%, *p* = 0.021). In both cases, the NRD was able to restore these strain values to their intact levels (*p* ≥ 0.192).

**Discussion:** The ability of the NRD to restore IVD biomechanics and some strain types to intact state levels supports nucleus replacement surgery as a viable treatment option. The DVC-MRI method used in the present study could serve as a useful tool to assess future NRD designs to help improve performance in future clinical trials.

## 1 Introduction

Low back pain (LBP) is the leading cause of disability, inflicting substantial economic burden to public health systems worldwide (Balagué et al., 2012; Hoy et al., 2014; GBD, 2019 Diseases and Injuries Collaborators et al., 2020). LBP is a complex and poorly understood condition that has been linked to biochemical and structural changes to the intervertebral disc (IVD). Degenerate disc disease (DDD) is one condition that has been linked to LBP (Bendix et al., 2008; De Schepper et al., 2010), and is thought to stem from an imbalance in the turnover of the extra-cellular matrix within the central region of the IVD, the nucleus pulposus (NP) (Millward-Sadler et al., 2009; Adams and Dolan, 2012; Mayer et al., 2013). Herniations are structural failures of the IVD in which the NP is extruded and impinges the adjacent nerves (Heo et al., 2017). Surgically, herniations are treated by removing the protruding tissue in a procedure called discectomy. Care must be taken when deciding how much material should be removed since limited discectomy can lead to re-herniation and aggressive discectomy can lead to accelerated degeneration (Heo et al., 2017; Martens et al., 2018; Rogerson et al., 2019).

Nucleus replacement devices (NRDs) have been proposed as a treatment for DDD as well as a means of mitigating the effects of aggressive discectomies (Bao and Hansen, 2002; Ray, 2002; Ahrens et al., 2009; Balsano et al., 2011). They aim to restore disc height and function, preserve the annulus fibrosus (AF), and alleviate pain (Bao and Hansen, 2002). Although more than 10 clinicals trials have been conducted on different NRDs, no device is currently used routinely in clinic due to high failure rates, with expulsion, subsidence, and loss of disc height observed during clinical trials (Bao and Hansen, 2002; Bertagnoli and Kumar, 2002; Ray, 2002; Di Martino et al., 2005; Boyd and Carter, 2006; Kettler et al., 2007; Wardlaw et al., 2008; Ahrens et al., 2009; Berlemann and Schwarzenbach, 2009; Balsano et al., 2011; Ziegler et al., 2019; Du et al., 2020). Understanding the interactions between NRDs and surrounding tissues may lead to improved NRD designs.

NP removal (nuclectomy) has been shown to alter disc biomechanics, and NRD implantation has been shown to restore IVD strains through computational studies (Rundell et al., 2009; Strange et al., 2010; Yang et al., 2021). However, the internal NRD-IVD interaction data from computational models are difficult to validate due to the challenges associated with collecting experimental data. Internal IVD deformations have been quantified *in vitro* (Meakin et al., 2001; Tsantrizos et al., 2005; Costi et al., 2007), using approaches that disrupt the IVD’s structural integrity and only provide discrete measurements at specific locations. More recently, advancements in image registration techniques have enabled the measurement of full-field strains on volumetric images (such as MR) *in vitro* and *in vivo* (Showalter et al., 2014; Yoder et al., 2014; Tavana et al., 2020; Menon et al., 2021; Tavana et al., 2021; Tavan et al., 2023). Although O’ Connell et al. (2011) and Claeson et al. (2019) investigated the effects of nuclectomy on mid-transverse disc and AF strains, respectively, these techniques have not been applied to measure entire IVD strains following nuclectomy and NRD insertion. An improved understanding of how nuclectomy affects internal disc strains, and how NRDs interact with surrounding tissues, may help identify root causes of NRD failure and provide target performance criteria for future NRDs.

The aim of this study was to use a previously validated image registration technique (Tavana et al., 2020; Tavana et al., 2021) to evaluate internal 3D strains of entire IVDs in three states: intact, post-nuclectomy and post-treatment with NRD. A secondary aim was to determine whether this technique is a useful tool to assess NRD designs *in situ*.

## 2 Materials and methods

### 2.1 Specimen preparation

Seven human lumbar functional spinal units (FSUs), each consisting of half vertebrae-disc-half vertebrae, from four donors with an average age of 42 ± 18 years were used (Table 1). Ethical approval was obtained from the Imperial College Tissue Bank Ethics Committee (approval number: 17/WA/0161). Soft tissues were removed with care taken to not damage the IVD. Posterior elements were removed by cutting through the pedicles ∼2 mm from the edge of the vertebral body. Prior to fixing specimen position within the non-magnetic MRI compatible pots, fluoroscopic images (InsightFD Mini-C-Arm, Fluoroscan, MA) were acquired to ensure that the disc’s mid-transverse plane was horizontal. Superior and inferior vertebrae were then secured using polymethyl-methacrylate. Specimens were regularly sprayed with 0.15 M phosphate buffered saline (PBS). When not being tested, specimen were wrapped in PBS-soaked paper-towel and enclosed in a double-bagged zip-lock bag.

**TABLE 1:** Specimen details. Degeneration grades (Pfirrmann et al., 2001), are rounded averages of rankings made by three observers.

Specimen number	Sex	Age (years)	Level	Degeneration grade	Nuclectomy technique
1	F	22	L4-L5	3	Auto-shaver
2	M	38	L2-L3	3	Rongeurs
3	M	38	L3-L4	3	Auto-shaver
4	M	42	L1-L2	2	Rongeurs
5	M	42	L2-L3	2	Auto-shaver
6	M	65	L3-L4	3	Rongeurs
7	M	65	L4-L5	3	Auto-shaver

### 2.2 Procedure to measure IVD stiffness

To compare stiffnesses between states, specimens were axially compressed at 1 Hz between 50 N and 1 kN for five cycles (Instron, Model 5866, High Wycombe, United Kingdom). Consistent with previous studies (Wilke and Claes, 1998; Holsgrove et al., 2015; Newell et al., 2019), preliminary work showed five cycles to be sufficient for a repeatable force-displacement response. Data from the fifth cycle was used to calculate toe and linear region stiffnesses by finding tangents between 50–200 N and 500–900 N, respectively (Bezci and O’Connell, 2015; Newell et al., 2020). Following this loading, specimens were unloaded and allowed to rest for 5 min whilst wrapped with PBS-soaked paper-towel. Preliminary work showed that 5 min was sufficient for 99% height recovery.

### 2.3 Unloaded and loaded MRI scans

To calculate internal IVD strains, MRIs of unloaded (reference) and loaded (deformed) discs were required. Each specimen was scanned with an ultra-high field 9.4T MRI system (Bruker BioSpec, Ettlingen, Germany) using a T2-weighted RARE sequence (in-plane resolution = 90 µm^2^ × 90 µm^2^, slice thickness = 800 µm, slice gap = 560 µm, ∼20 min scan time) in all three stages, and in both unloaded and loaded conditions. This scan sequence has been previously used and validated for this application by Tavana et al. (2020).

A bespoke MRI-compatible loading rig fixed and held specimen displacement during scanning whilst also ensuring repeatable positioning within the MRI bore (Tavana et al., 2020; Tavana et al., 2021). The desired load was achieved by compressing specimens using the materials testing machine. The displacement of the specimen under the desired load was then fixed by tightening nylon nuts on the three nylon threads that passed through the top and bottom pots of the loading rig. For the unloaded images, specimens were compressed to a nominal 50 N to ensure pot-loading rig contact, whilst for the loaded scans, 1 kN was applied. 1 kN approximately represents the average axial force experienced when standing relaxed or sitting with a straight back (Wilke et al., 1999). Preliminary experiments showed that 1.5 h of resting at the 1 kN level of fixed displacement was sufficient to ensure an IVD relaxation rate of < 1 N/min before MRIs were acquired. Minimising relaxation rate was assumed to reduce internal tissue movement during imaging. These steps were repeated such that unloaded and loaded images could be captured for each specimen in their intact, post-nuclectomy and post-treatment states. Additional preliminary work using potentiometers attached to the top and bottom of the rig to assess deformation of the nylon screws holding the specimen demonstrated that the rig maintained the desired displacement with 93% accuracy over 2 h, with height changes attributed to deformation of the pot material radially bulging over time. To counter this, new 3D printed pots used to hold the specimens were made for each specimen.

### 2.4 Disc degeneration grading and disc height measurements

Each IVD was graded by three observers from the unloaded intact MRIs. Using a 9.4T MRI scanner enabled detailed interior IVD features to be captured, including the NP:AF boundary and AF bulging direction. The standard Pfirrmann algorithm (Pfirrmann et al., 2001) was used to grade disc degeneration, however these internal features gave additional information that was also considered through the evaluation process. IVDs exhibiting outward AF bulging and a distinct AF:NP boundary were categorised as grade I or II based on the signal characteristics as proposed by Pfirrmann et al. (2001). Grade III was designated for IVDs with a clear AF:NP boundary, which also displayed the initiation of inner bulging of the AF or no bulging at all. Grade IV was attributed to discs where the AF:NP boundary was indistinct, accompanied by inner bulging of the AF, and the presence of a fibrous NP region. In cases where the disc space had collapsed, a grade of V was assigned. Specimens were deemed suitable if 1) endplate damage or AF tears were not visible on MRIs, 2) the level of degeneration was ≤ Pfirrmann grade 4 (Pfirrmann et al., 2001), and 3) that disc height was ≥ 5 mm (Coric and Mummaneni, 2008).

Mimics (Materialise HQ, v.19.0, Leuven, 97 Belgium) was used to measure the central disc height from the unloaded, intact, coronal plane MRI slice with the largest lateral disc width on the corresponding axial slice. Three repeat measurements were made per slice, and six further height measurements were obtained from the neighbouring two slices. The disc’s centre in each slice from which height measurements were made was determined using the technique described by Rahman et al. (2022). The average of the nine measurements was then taken as the mean disc height.

### 2.5 Nuclectomy

Nuclectomy was performed using either an automated-shaver (Nucleotome*®*, Clarus Medical, MN) or rongeurs. This was to investigate whether different nuclectomy techniques affect the internal strain distributions.

Preliminary work showed, in accordance with O’Connell et al. (2011), that wrapping the specimens with PBS-soaked paper-towed overnight at 4°C enabled disc height to be fully restored. Prior to nuclectomy, specimens were allowed to equilibrate at room temperature before fixing their displacement at the 50 N load level. A spinal surgeon (JB) removed the nucleus material using one of the two techniques described in the following sections. The tissue extracted was dehydrated in a fume-hood for 48 h so that dry masses could be weighed. Following nuclectomy, MRIs were acquired, and specimen were allowed to recover overnight.

#### 2.5.1 Nuclectomy using an automated-shaver

A #11 scalpel was used to make a small puncture into the AF such that the 1.5 mm guidewire could be inserted with the tip positioned centrally within the IVD (confirmed via sagittal and frontal plane C-arm fluoroscopy, InsightFD Mini-C-Arm, Fluoroscan, MA). A cannula and trephine were inserted over the guidewire to incise the AF. The trephine was replaced by the 3 mm diameter automated-shaver probe. As the probe was activated, NP material was collected in a cannister. The probe was moved in a fan shape and rotated to maximise tissue removal for at least 20 min until NP material could no longer be seen passing through the suction tubes for 2 min as described by Rahman et al. (2022).

#### 2.5.2 Nuclectomy using rongeurs

A triangular incision was made into the posterolateral AF such that rongeurs (3 mm diameter) could be inserted. NP material was gripped, torn, and extracted from the disc. The rongeurs continued to be re-inserted until the surgeon indicated that no further material was removed from the disc following three consecutive attempts (Rahman et al., 2022).

### 2.6 Post-treatment: NRD insertion

The NP void was filled with a string-like preformed hydrogel-based NRD previously developed by Synthes Spine LLP (Philadelphia, United States). The NRD was prepared at Imperial College London according to the formulation disclosed in the granted US Patent (US 8118874 B2) under the guidance of NS. Unconfined compression tests using the protocol developed by Cloyd et al. (2007) characterised the linear region stiffness of the NRD. The NRD was delivered into the IVD using a custom-built device consisting of a 10G needle attached to a Tygon^®^ tube and syringe. The needle was inserted through the annulotomy and the NRD was injected until internal resistance prevented any further material from being inserted. This technique has been described previously as haptic feedback (Dixon et al., 2021). Where NRD material was pushing back out of the annulotomy, thin forceps were used to re-insert loose ends. To ensure correct placement and that the cavity was filled, sagittal and frontal plane fluoroscopic images were acquired. Post-treatment MRIs were then captured.

### 2.7 Image processing and digital volume correlation

Digital Volume Correlation (DVC) is an image registration technique that calculates deformation by dividing volumetric images into sub-volumes called subsets. Patterns are tracked between the reference (unloaded) and deformed (loaded) MRIs. 3D binary region-of-interest masks were created using Mimics for each specimen through manual segmentation from the unloaded MRI to ensure only IVD tissue was analysed. Pre-processing was carried out using bicubic interpolation in the *z*-direction to transform the raw non-cubic voxels (90 µm^3^ × 90 µm^3^ × 1360 µm^3^) into 90 µm^3^ × 90 µm^3^ × 90 µm^3^ cubic voxels (ImageJ 1.8.0, National Health Institute, United States). The DVC-MRI protocol using DaVis (8.4.0, LaVision, Germany) developed by Tavana et al. (2020); Tavana et al. (2021) was then implemented. The displacement and strain errors of this method have previously been shown to not exceed 28 μm and 3000 microstrain respectively, in human cadaveric discs that have been tested using the same loading protocol (Tavana et al., 2020). Additionally, the NP and AF regions from each IVD were manually segmented so that strains from different disc locations could be compared. In the post-nuclectomy groups, the NP strains were omitted since there were no unique patterns present in the void region for DVC to track. The AF was further divided into anterior (A), posterior (P) and two lateral regions (L1 and L2), with the L2 region being nearest to the posterolateral annular incision.

### 2.8 Radial and circumferential strains

The strains of each voxel were transformed to a local radial-circumferential coordinate system, as described by Yoder et al. (2014). For each voxel, a line was projected from the centroid of the outline of the disc, through the voxel centre, and onto the outline. At the intersection of this line and the outline, the tangential and normal directions were used to define the circumferential and radial basis vector directions, respectively, for the local coordinate system of the voxel.

### 2.9 Statistical analysis

A D’Agostino-Pearson test was used to assess normality prior to conducting statistical analyses, and Levene’s test to assess homoscedasticity. Where normality and equal variance was violated, a Kruskal-Wallis test was used to conduct group comparison. Differences in strains between stages were assessed using two-way ANOVA followed by Tukey’s multiple comparison test. Significance was set at *p* = 0.05. All statistical analysis were performed using GraphPad Prism 9.4.1 (GraphPad Software, San Diego, California United States).

## 3 Results

### 3.1 Disc height and stiffness

**FIGURE 1:**
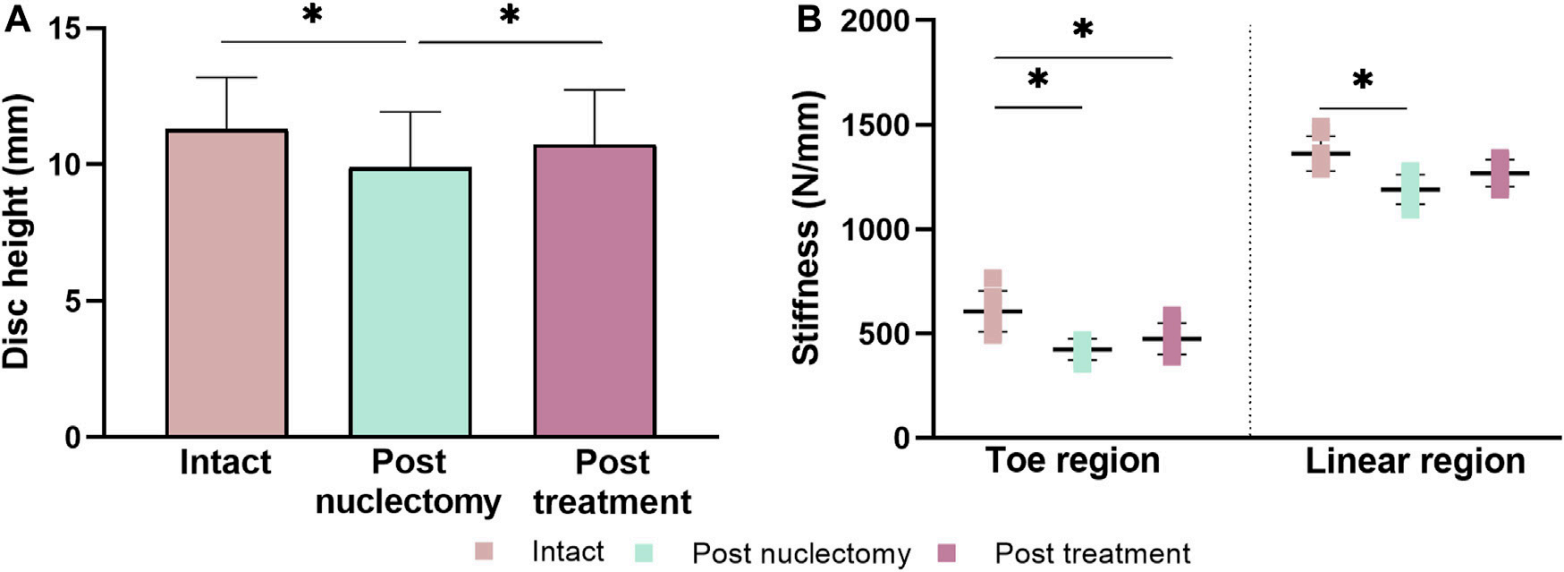
**(A)** Bar chart of mid-coronal disc heights measured from the unloaded state MRIs in intact, post-nuclectomy and post-treatment states. **(B)** Stiffness dot diagrams for toe and linear regions. Error bars represent standard deviations. Asterisk denotes statistical significance (*p* < 0.05).

Due to endplate fracture, post-treatment data from specimen #4 were excluded. Loading to 1 kN resulted in similar (*p ≥* 0.092) disc height reductions in all states; 0.9 ± 0.4 mm intact, 0.8 ± 0.4 mm post-nuclectomy, and 0.8 ± 0.5 mm post-treatment (Figure 1A). Compared to the intact unloaded state, disc height post-nuclectomy significantly reduced by 12.6% ± 4.4% (*p* = 0.004), whilst post-treatment recovered to within 5.3% ± 3.1% of the intact height (*p* = 0.095). There was no significant difference in the dry NP material removed using the automated-shaver (242 ± 84 mg) and using rongeurs (235 ± 2 mg), *p* = 0.32, nor were disc volumes significantly different between the two groups (nucleotome: 13.8 ± 3.9 cm^3^, rongeurs: 15.9 ± 4.7 cm^3^, *p* = 0.628).

Post-nuclectomy, both the toe and linear region stiffnesses dropped relative to the intact state (29.0% ± 5.4% and 12.3% ± 7.0%, respectively) (*p* ≤ 0.01, Figure 1B). Post-treatment, the linear region stiffness was restored to within 7.8% ± 5.9% of the intact state (*p =* 0.065), however, the toe region stiffness was 21.7% ± 8.7% lower than the intact state (*p =* 0.007). The NRD was significantly stiffer than the native NP data reported in Cloyd et al. (2007) (144.0 ± 14.6 kPa versus 0.62 ± 0.15 kPa).

### 3.2 Effect of nuclectomy technique on strain distribution

Due to tensile axial strains present in specimen #4 (assigned to the rongeurs group), the data was considered an outlier and removed from the analysis. In the intact state, there were no differences in any strain type between specimens assigned to the automated shaver or rongeurs groups (Supplementary Figure S1). Post-nuclectomy, the technique did not affect the average AF axial strain (−13.6% ± 4.1% and −12.2% ± 2.7%, respectively). Additionally, pairwise comparison of the average strains in each region of the IVD between the post-treatment and the intact state showed that the NRD restored all strain values to within 6.0% ± 2.7% of the intact state, irrespective of nuclectomy technique (*p >* 0.118).

### 3.3 Average strains

**FIGURE 2:**
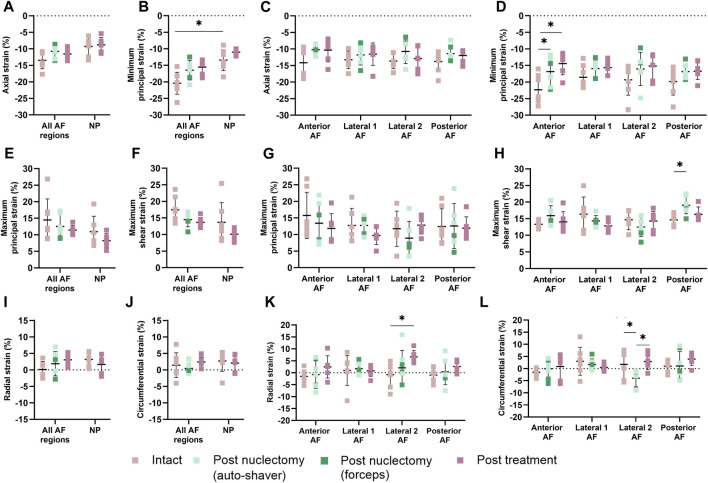
**(A, B, E, F, I, and J)** Mean (SD) strains in nucleus (NP) and **(C, D, G, H, K, and L)** annulus (AF). The AF was split into anterior, posterior and lateral (1 and 2) regions, with the posterolateral annular incision at the posterior and lateral-2 AF boundary. **(A, C)** Axial strain, **(B, D)** minimum principal strain, **(E, G)** maximum principal strain, **(F, H)** maximum shear strain, **(I, K)** radial strain and **(J, L)** circumferential strains. A single asterisk denotes statistical significance (*p <* 0.05). Note the annulotomy was located close to the boundary of the Posterior AF and Lateral 2 AF regions.

Post-nuclectomy groups were pooled due to the non-significant differences between the disc sizes, stiffnesses, and NP removal volume. Average axial strain within the NP region significantly increased from −9.3% ± 2.3% intact to −15.3% ± 9.3% following nuclectomy (*p* = 0.010). Post-treatment, the average axial NP strains were similar to intact (−8.8% ± 2.2%, *p* = 0.593) (Figure 2A). The average AF strains were homogeneously distributed throughout all three stages (average values in neighbouring regions within 5.3% of each other) (Figures 2C, D, G, H, K, and L).

In the intact state, the average minimum principal AF strain was significantly higher than that of the NP (−20.4% ± 3.3% vs. −13.4% ± 3.2%, *p* = 0.009). The regional average AF minimum principal strain values were not affected post-nuclectomy and post-treatment (*p* ≥ 0.079), except for the anterior region which showed lower strains post-nuclectomy (−16.8% ± 4.5%) and post-treatment (−14.4% ± 3.4%) compared to the intact state (−22.3% ± 4.4%, *p ≤* 0.03*,* Figure 2D). The maximum shear strains in the posterior AF were significantly increased post-nuclectomy (19.4% ± 2.6%, *p* = 0.021) compared to the intact state (14.6% ± 1.7%). Post-treatment, the shear strains were restored (16.3% ± 2.2%, *p* = 0.607, Figure 2H). On average, post-nuclectomy and post-treatment radial AF strains were not significantly different relative to intact (*p ≥* 0.108, Figure 2I). However, average radial strain within the lateral-2 AF region significantly increased post-treatment (6.6% ± 2.7%) compared to the intact state (−0.8% ± 5.0%, *p* = 0.005) (Figure 2K). Circumferential strains within the AF lateral-2 region significantly decreased post-nuclectomy (−4.0% ± 3.4%) compared to the intact state (1.7% ± 6.0%, *p* = 0.013). Post-treatment, circumferential strains were restored to values similar to the intact state (*p* ≥ 0.192, Figures 2J, L).

### 3.4 Peak strains

**FIGURE 3:**
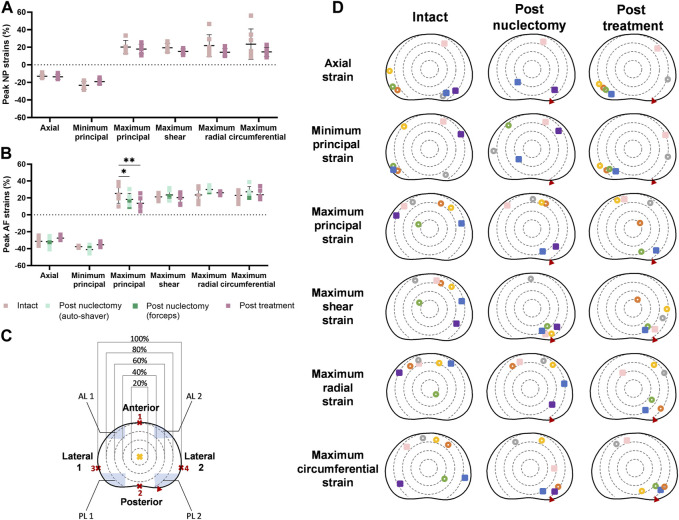
Dot diagrams of peak strains in the **(A)** nucleus (NP) and **(B)** annulus (AF) regions. **(C)** The disc has been subdivided into circular regions following a method described by Rahman et al. (2022). The geometrical centre of the disc (yellow cross) was assumed to be at 50% of the largest anterior-posterior width (points 1 and 2). The diameter of the circular regions was determined by calculating 20, 40, 60, and 80% of the largest lateral width of the disc measured between points 3 and 4. The posterolateral (PL) and anterolateral (AL) regions are defined by the blue shaded regions. The largest width of the regions is 30% of the distance between point 3 and 4 and the largest height is 20% of the distance between point 1 and 2. Error bars represent standard deviations. A single asterisk denotes statistical significance (*p <* 0.05). **(D)** Peak strain locations plotted on a transverse plane slice of a disc. Each specimen is represented by a colour. Hollow circles and filled squares represent specimens from the automated shaver and rongeurs group, respectively. Red triangles indicate the location of the annular insult through which nuclectomy was performed. In the post-nuclectomy group, peak strains which were located in the NP region (40% of the anterior-posterior and lateral width) were omitted.

In the intact state, peak axial NP region strain (−12.9% ± 2.8%) was similar to the post-treatment state (−13.6% ± 2.9%, *p =* 0.402). The average peak maximum principal AF strain significantly reduced post-nuclectomy relative to intact, but was not restored post-treatment, from 25.2% ± 11.5% to 18.3% ± 7.1% (*p =* 0.042), and 13.6% ± 7.6% (*p =* 0.001), respectively (Figure 3B). Peak strain magnitudes were not significantly different for radial or circumferential strains in either the NP or AF (Figures 3A, B).

In the intact state, peak strain locations were more common in the peripheral regions of the AF with 90% being within the 80th and 100th percentile from the geometric disc centre (Figure 3D). In the intact state, 70% of axial peak strains occurred within the posterolateral regions whilst 85% of the maximum principal strains occurred in anterolateral regions. Additionally, 70% of peak shear strains were within the 60th and 80th percentile bands in the anterolateral and anterior regions. However, post-nuclectomy, 70% of specimens showed peak shear strains within the 60th and 80th percentile bands of the posterolateral-2 region (annulotomy region). Post-treatment, 85% of peak axial and minimum principal strains were in posterolateral regions (Figure 3D). In the intact state, 85% and 57% of peak radial and circumferential strains occurred within the anterior AF, respectively (Figure 3D). Post-nuclectomy, 70% of peak radial strains remained within the anterior AF (within the 60th and 80th percentile bands), whilst 70% of peak circumferential strains were located in the lateral-2 and posterolateral-2 regions, proximal to the annular incision. Post-treatment, peak radial and circumferential strain locations were not restored, with 60%–80% of strains found in the lateral-2 and posterolateral-2 AF regions, respectively.

### 3.5 Endplate deformation and inner annulus bulging

**FIGURE 4:**
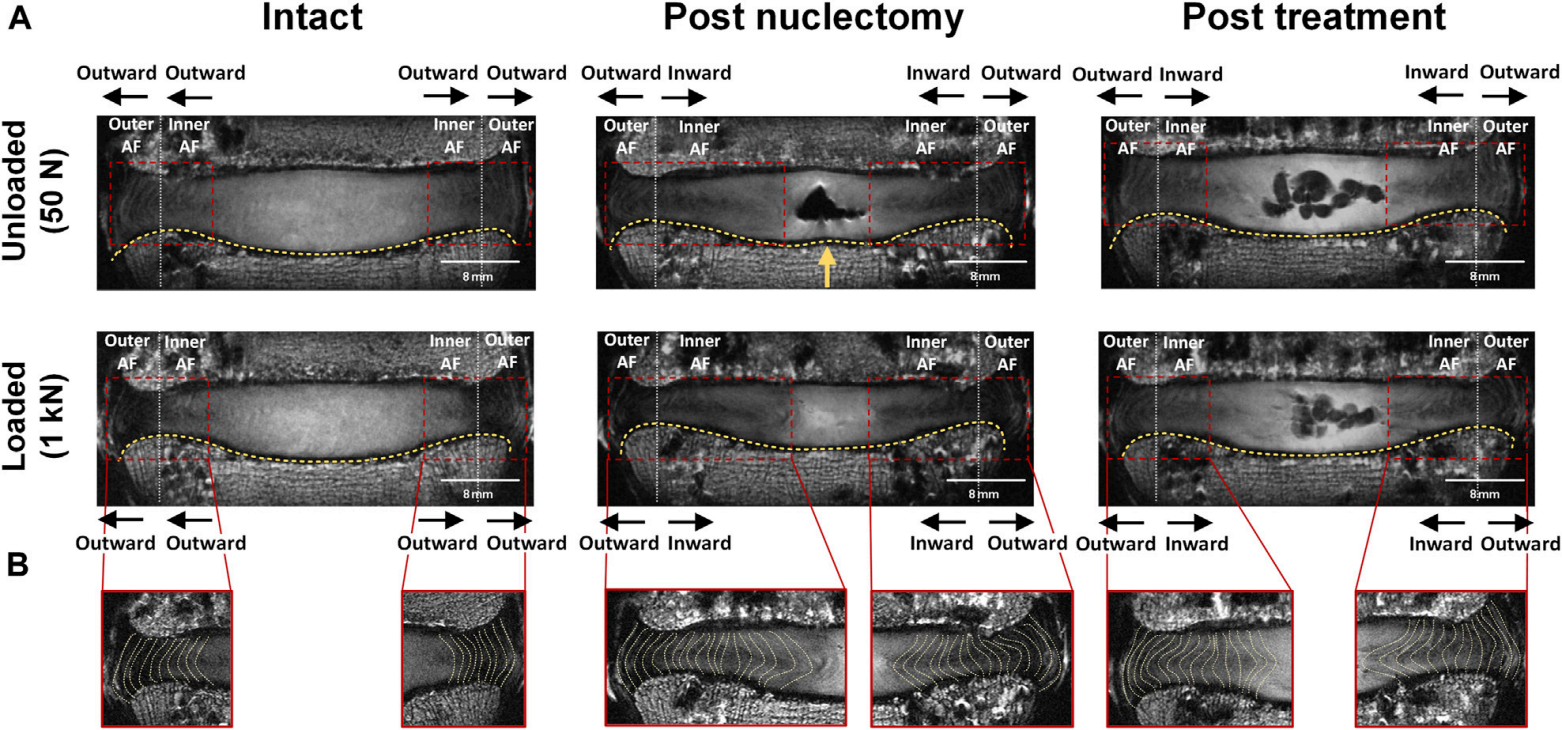
**(A)** Mid-coronal MRI slice of a typical disc both unloaded and loaded during the three surgical states. The yellow arrow indicates the location at which the curvature of the endplate differed from that seen in the unloaded intact state (slight upward deformation). The red box identifies the AF region which has been split by the white vertical line to define inner AF and outer AF regions. Black arrows indicate the direction of AF bulging. **(B)** Shows the left and right lateral AF regions with the yellow dashed lines delineating the changes in lamellae morphology during the three states of the nucleus replacement surgery. The change in AF bulging direction as seen in this specimen only occurred in this and one other specimen out of seven.

Endplate profiles were visually inspected by comparing the loaded and unloaded coronal view MRIs in each surgical state. In two instances endplate deformation was observed adjacent to the main site of NP tissue removal in the unloaded state only (indicated by a yellow arrow in Figure 4A). In one specimen (not shown in Figure 4A), loading resulted in endplate bulging into the vertebra. Post-treatment, this specimen (#4) failed due to an endplate fracture. Axial loading, nuclectomy, or NRD implantation did not affect the bulging direction of the inner AF in most specimens (see black arrows in Figure 4A). In two specimens, the inner AF’s bulging direction changed from outwards when intact to inwards post-nuclectomy. One of these specimens is shown in Figure 4B.

## 4 Discussion

This is the first study to non-invasively measure internal strains within whole IVDs through three stages of nucleus replacement surgery. DVC-MRI was explored as a potential tool to assess interactions between NRD and surrounding tissues, in turn providing insight into the suitability of NRDs for clinical use.

Mid-disc height loss in the intact discs following 1 kN of compression (0.9 ± 0.4 mm) was similar to the ranges reported by O’Connell et al. (2011) and Claeson et al. (2019) (0.46–0.84 mm and 0.9–1.43 mm, respectively). O’Connell et al. (2011) compressed their specimens to 1 kN, whilst Claeson et al. (2019) compressed their specimens to 10% axial strain, but the fact that disc height changes were similar means that strains can reasonably be compared. On average, 238 ± 69 mg of dry NP material was removed in this study leading to a significant loss of disc height (12.6% ± 4.4% lower than intact, *p* = 0.0004). However, Claeson et al. (2019) and O’Connell et al. (2011) reported non-significant loss of disc height post-nuclectomy. This can be attributed to both Claeson et al. (2019) and O’Connell et al. (2011) rehydrating specimens for at least 8 h following nuclectomy. This rehydration and redistribution of the remaining NP material may have reduced the effect of nuclectomy on disc height loss. In this study, the discs were not rehydrated so that the immediate effects of nuclectomy could be investigated, the material properties of the disc tissues were unaffected, and there was consistency in MRI signals between states.

The observed decrease in toe and linear region stiffness following nuclectomy (29% ± 5.4% and 12.3% ± 7.0%, respectively, *p* ≤ 0.01) is within the 3%–35% range reported by previous studies (Cannella et al., 2008; Showalter et al., 2014; Rahman et al., 2022). Post-treatment, both disc height and linear region stiffnesses were restored to values similar to intact (Figure 1). However, the toe region response was not restored, suggesting that the NRD investigated cannot currently mimic the physiological disc’s response to lower loads (<200 N). It was expected that AF strain, particularly axial strain, would significantly increase post-nuclectomy, however, all regional AF strains were within 5.3% of each other, irrespective of state (Figure 2). This relatively homogeneous AF strain distribution agrees with the *in vitro* results reported by previous studies (O’Connell et al., 2011; Claeson et al., 2019). This homogeneity is likely due to the pure axial compressive load applied. Post-treatment, all strains were restored to within 6.0% ± 2.7% of intact, suggesting that this NRD has the potential to reverse the effects of nuclectomy under axial compression. However, in the two instances where inner AF bulging was observed (Figure 4), the NRD was unable to reverse the AF bulging direction. This may be due to insufficient NRD volume being delivered into the IVDs. Improving the delivery method might enable more NRD volume to be inserted, though this may trade-off with an increased risk of expulsion (Ray, 2002; Allen et al., 2004; Balsano et al., 2011).

In healthy IVDs, NP pressure causes AF collagen fibres to experience increasing tensile strains as a function of increased distance from the disc’s centre (Żak and Pezowicz, 2021). This potentially provides an explanation for the peak maximum principal strains in the intact state being predominantly in the AF periphery (Figure 4C). 70%–85% of all peak maximum and shear strains in the intact state were found within the 60th-80th percentile of the anterior AF (Figure 3C), possibly explaining the high incidence of peripheral tears within this region as observed by Osti et al. (1992). In coronal and sagittal views, peak strains were observed at the AF-endplate interface (for shear and maximum principal strains), or in the AF (axial and minimum principal strains), similar to the findings of Tavana et al. (2021) and O’Connell et al. (2011).

Contrary to expectations, post-nuclectomy AF strains did not significantly increase compared to the intact state (Figure 2), significantly decreasing for minimum principal strain (intact −22.3% ± 4.4%, post-nuclectomy −16.8% ± 4.5% *p ≤* 0.03, Figure 3D). This may pertain to the inability of the DVC software to detect residual strains occurring between intact and post-nuclectomy in the unloaded (50 N) state, i.e., displacement vectors between intact and post-nuclectomy could not be calculated. Furthermore, the loss of disc height after NP removal creates a shorter baseline height of which to measure compressive strain against (Yoder et al., 2014). The AF strain values between stages are similar as the ratio between displacement of the IVD under loading and the unloaded height remained similar, e.g., post-nuclectomy the (shorter) IVD displaces less than in intact (taller) IVD, demonstrating the role of the NP in maintaining disc height under load. Future NRD designs should ensure that disc height can be maintained under load, restore both toe region and linear region IVD stiffness, repressurise the AF, and ensure homogeneous strain distributions across the IVD, assuming pure axial compression.

**FIGURE 5:**
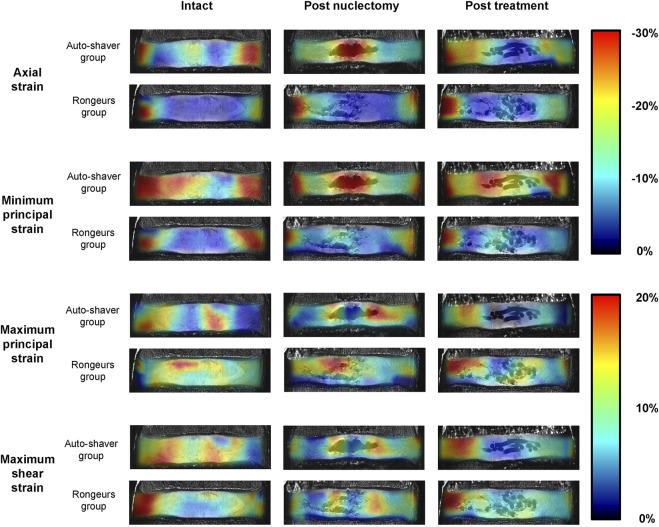
Mid-coronal views of 9.4T MRIs of two typical specimens (one from the automated shaver nuclectomy group and one from the rongeurs group). The MRIs in the unloaded state have been superimposed by the axial, minimum principal, maximum principal, and maximum shear strain distribution maps to demonstrate changes through the three surgical stages. Note that in the post-nuclectomy group, the strain maps cover the void region, but these regions were excluded from the analysis.

**FIGURE 6:**
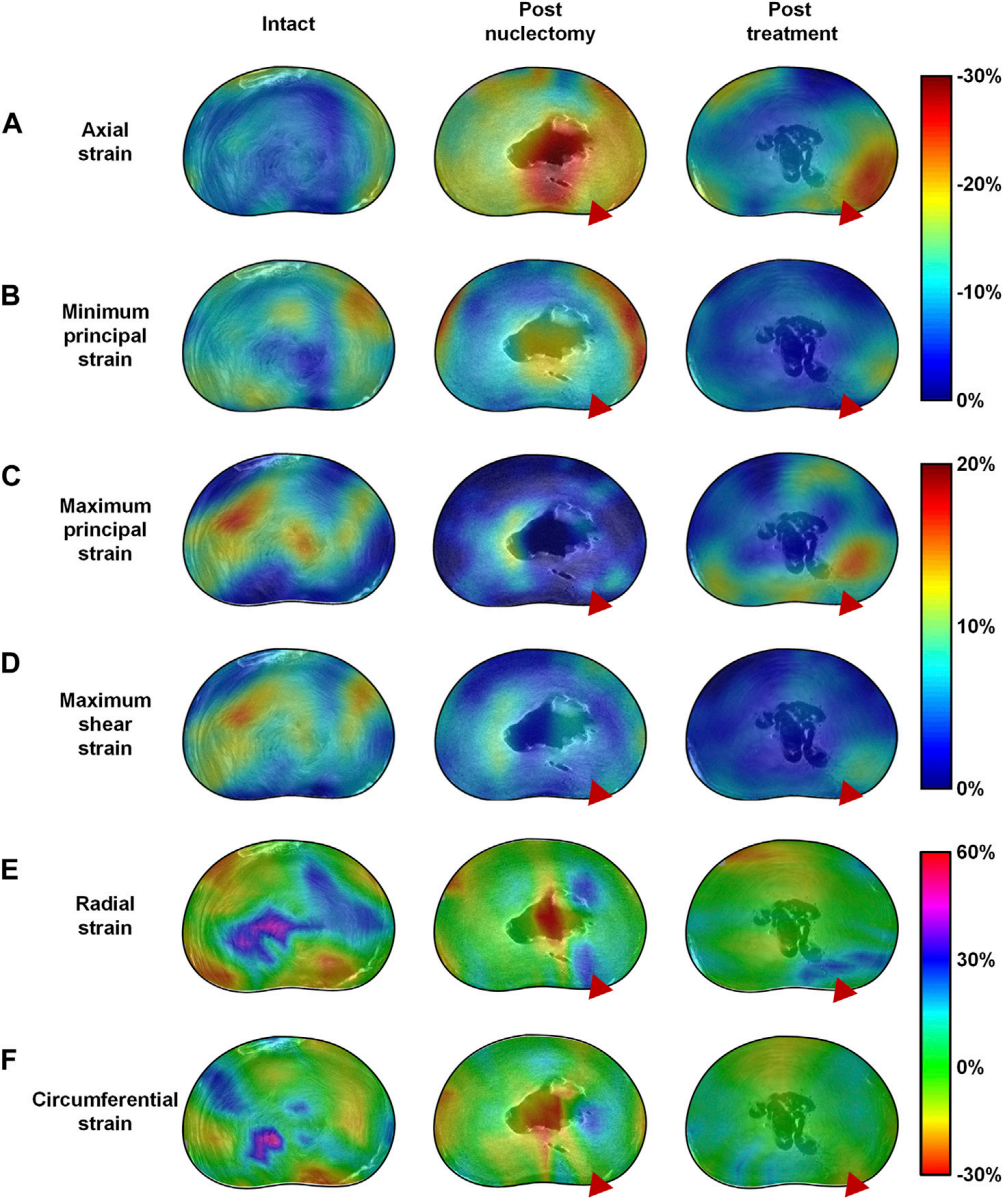
Mid-transverse ultra-high resolution MRI slice of a typical disc in the intact, post-nuclectomy and post-treatment states superimposed with the mid-disc axial view 3D strain distribution maps of **(A)** axial, **(B)** minimum principal, **(C)** maximum principal, **(D)** maximum 3D shear strains, **(E)** radial strains and **(F)** circumferential strains. In the disc pictured above nuclectomy was performed using the automated shaver, following which the cavity was filled with a nucleus replacement device as can be seen in the centre of the disc in the post-treatment MRIs. The red triangle indicates the location of the annular insult through which nuclectomy was performed. Note that in the post-nuclectomy group, the strain maps cover the void region, but these subsets were excluded from the analysis.

Post-nuclectomy and post-treatment, 70% of peak shear strains shifted towards the posterolateral region where the annulotomy was performed, significantly increasing the shear strain within the region to 19.4% ± 2.6% (from 14.6% ± 1.7% intact). This may be due to redistribution of adjacent tissues to close the AF incision site as the IVD was loaded. Additionally, the redistribution of AF tissue around the NP cavity resulted in regions of high maximum principal and shear strains in the AF tissue immediately surrounding the NP cavity (Figures 5, 6). These findings are relevant to disc herniation treatments in that they show that aggressive discectomy or nuclectomy can create regions of high strain that could result in tears or accelerated degeneration. Increased IVD tissue strains have been linked to upregulated inflammatory responses that can lead to disc degeneration (Gawri et al., 2014), which may explain why aggressive discectomy or nuclectomy alone can accelerate disc degeneration. Preventing regions of high strains around cavities or incision locations may be important design specifications for NRDs and AF closure systems.

Post-treatment, disc heights were restored to within 5.3% ± 3.1% of the intact state. All strains significantly affected by nuclectomy were restored close to intact values, except for the peak maximum principal strain which remained lower (Figure 3B). Additionally, from Figure 2 it can be observed that, although not statistically significant, all strains were lower in magnitude post-treatment relative to the intact state. These findings have two potential implications. Firstly, the lowered peak tensile strains in the AF might be advantageous in that the AF is less likely to develop tears. Secondly, it is known that both tensile and compressive strains in the AF are required to stimulate IVD cells and extra-cellular matrix synthesis (Gilbert et al., 2010), but the long-term effects of lowered strains experienced by the AF are unknown, and could potentially lead to accelerated degenerative changes and delamination. Ideally, future NRDs should restore all components of AF strains, and in all modes of loading including axial compression, as was investigated in the present study.

When using the automated shaver, material was removed homogeneously from the centre of the disc, whilst when using rongeurs, disc material was torn resulting in uneven tissue removal. Different strain magnitudes and distributions were therefore observed between the two groups (Figure 5, Supplementary Figure S1), but this could not be quantified statistically due to the failure of one sample. However, rongeurs have previously been shown to disrupt AF integrity, thus should be used with caution in this regard (Rahman et al., 2022). Post-treatment, all average AF strains were restored to within 6.0% ± 2.7% of intact, irrespective of nuclectomy technique, suggesting that the technique may not affect the restoration of post-treatment strains. Further studies with a larger sample size, different NRD types, and loading modalities are required to confirm these initial findings.

Radial and circumferential strains represent AF bulging in response the hydrostatic pressure of non-degenerate NP (Seroussi et al., 1989). However, upon herniation or annular insult, this tension is disrupted, which is speculated to accelerate local tissue degeneration (Michalek et al., 2012). Thus, the AF must be able to withstand the radial pressure exerted from the NRD and its insertion procedure. In the lateral-2 AF region (proximal to the annular insult), radial strain was not restored post-treatment (6.6% ± 2.7%, *p =* 0.005, Figure 2L). Similarly, 70% of peak circumferential strains aggregated in the annulotomy region, which remained post-treatment (Figure 3D). This suggests that the annulotomy procedure induces an artefact when assessing the efficacy of an NRD to reinstate physiological IVD strains. The location of peak strains in the anterior region contrasts with Yoder et al. (2014), where a gradual decrease of strain magnitude from inner to outer AF was reported. However, average strain magnitudes in the present study were within the ranges of Yoder et al. (2014) (1.0% ± 2.4% versus −0.6% ± 4.2% (radial), 1.2% ± 1.3% versus 1.4% ± 3.8% (circumferential). More data characterising IVD radial and circumferential strains using image registration techniques would provide a more comprehensive understanding of the distributions of these strain types, and the effect of annulotomy alone versus the full nuclectomy procedure.

DVC-MRI has been shown to have promise as a tool to assess NRD devices. It has allowed complex interactions between NRDs and surrounding tissues to be investigated without disrupting the IVD’s integrity beyond what occurs during a nucleus replacement surgery.

A strength of this study is that the age range [up to 70 (Ahrens et al., 2009)], disc heights [>5 mm (Coric and Mummaneni, 2008)] and medical history [absence of spinal conditions (Coric and Mummaneni, 2008; Ahrens et al., 2009)] of the specimens used in this study are reflective of the requirements needed to be eligible for NRD treatment. However, inherent with studies of this kind, the sample size (*n* = 7), limited the generalisation and extrapolation of the results, and the range of lumbar levels included in this study may have introduced greater intra-group variation, although the repeated measures design is expected to control for this variation. Failure of a specimen (#4) post-treatment, potentially a result of preexisting microfractures in the superior endplate and the erroneous strain patterns post-nuclectomy*,* did not allow the significance of the difference in strains between the NRD-treated groups to be determined (automated shaver vs. rongeurs), and thus does not allow robust conclusions to be drawn regarding the impact of nuclectomy technique on IVD strain. Additionally, specimens were exposed to pure axial compression only, which despite being the major load direction when standing (Rohlmann et al., 2014), it is known that both load and strain distributions depend on the loading modality (Tavan et al., 2023; O’Connell et al., 2011). Therefore, future studies to investigate the use of DVC-MRI to better understand internal disc interactions with the NRD in more complex loading environments such as flexion/extension or lateral bending are recommended, as are further experiments to determine whether internal strains are sensitive to NRD shapes and stiffnesses. Additionally, a ramp loading profile to investigate dynamic changes in strain distribution would have been of interest. However, this was not possible due to inherent limitations of MRI image acquisition, but may be possible with other dynamic imaging modalities. Lastly, if DVC-MRI is included as an NRD assessment, a large number of specimens should be used and care must be taken when interpreting results since the analysis is based on static loading, at a single loading magnitude. Therefore, it is recommended that this tool is used as part of a suite of testing protocols to give a full picture of the effectiveness of a particular NRD design.

## 5 Conclusion

DVC-MRI enabled non-invasive quantification of complex 3D internal strains, demonstrating its potential value for assessing the interaction between NRDs and surrounding IVD tissues. The NRD tested in the present study showed potential to restore full-field circumferential and maximum shear AF strains to that of intact values, although failed to restore peak maximum principal strains and average minimum principal strain in the anterior AF. Biomechanical testing protocols of pre-clinical trial NRDs could be enhanced with the addition of image registration techniques to quantify internal IVD strains.

## Data Availability

The raw data supporting the conclusion of this article will be made available by the authors, without undue reservation.
