# Supplementary figures and images for: Quantifying internal intervertebral disc strains to assess nucleus replacement device designs: a digital volume correlation and ultra-high-resolution MRI study
Source: Front Bioeng Biotechnol. 2023 Oct 2;11:1229388. doi: 10.3389/fbioe.2023.1229388 (PMC10577660; doi:10.3389/fbioe.2023.1229388)

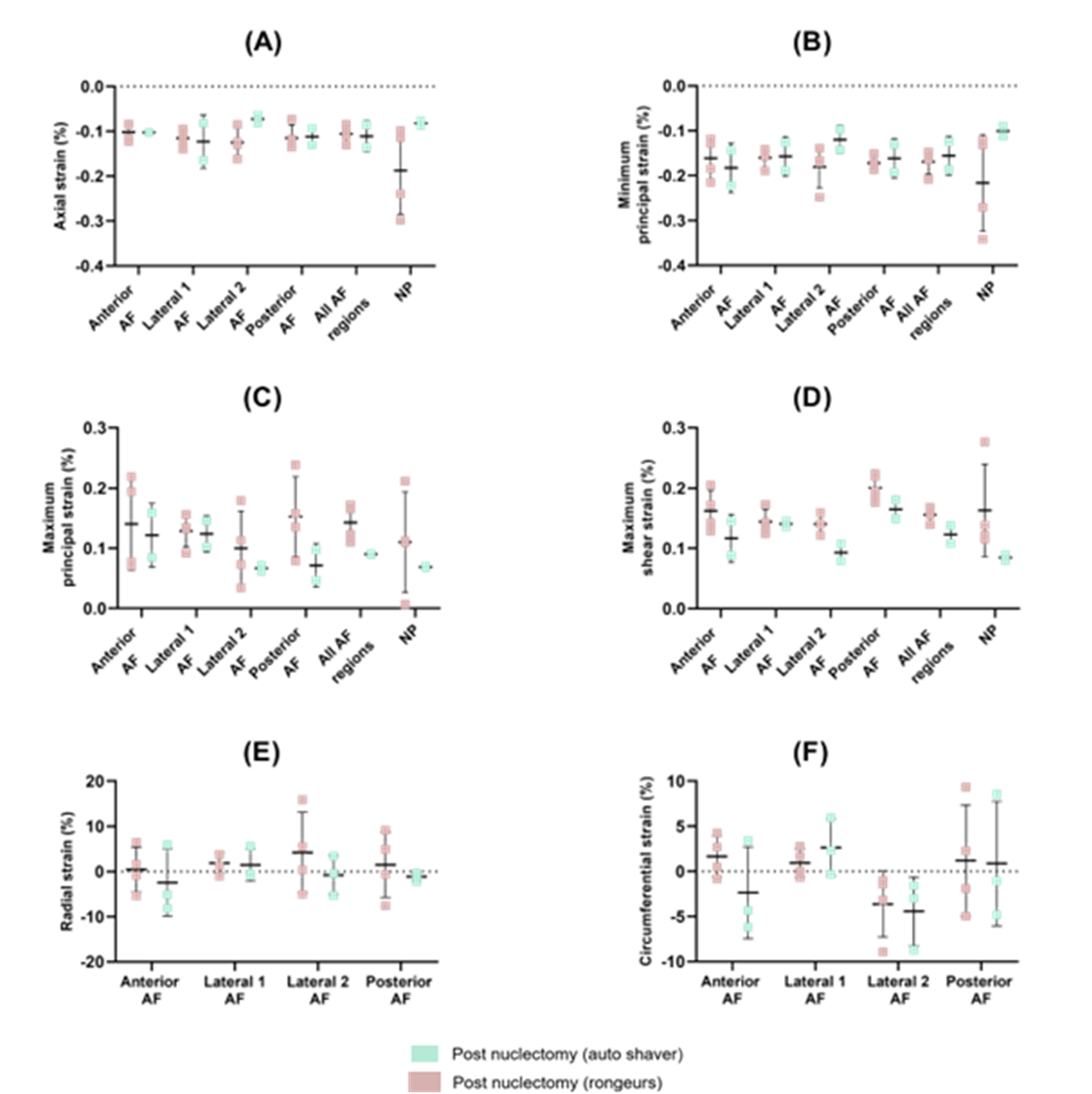

Supplement: Supplementary file 1 [file Image1.tif]
